# AdvanCE, a capsule endoscope delivery device, is effective in investigating a waterfall stomach

**DOI:** 10.1002/jgh3.12872

**Published:** 2023-02-01

**Authors:** Kazuya Miyaguchi, Yoshikazu Tsuzuki, Hiroyuki Imaeda

**Affiliations:** ^1^ Department of Gastroenterology Saitama Medical University Saitama Japan

**Keywords:** AdvanCE, capsule endoscopy, waterfall stomach

## Abstract

AdvanCE is a useful device in patients who cannot swallow capsule endoscopes, or when these remain in the patient's stomach without passing into the duodenum. A net or snare may be used to guide the capsule endoscope into the duodenum when the capsule endoscope becomes stuck in the stomach, but it may be difficult to guide the capsule into the duodenum in some cases, particularly in those of waterfall stomach. In such cases, AdvanCE is found to be an effective guidance tool.

## Graphical abstract

The capsule endoscopy was brought to the duodenum with AdvanCE using a guidewire as a landmark for the waterfall stomach.

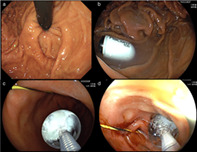

## Introduction

Examination of small bowel lesions is important in cases of unexplained anemia. The diagnostic rate of small bowel capsule endoscopy (CE) for unexplained iron deficiency is 47%.^1^

However, it is often abandoned in patients with dysphagia or difficulty passing CE through the pylorus.^2^ AdvanCE is a device that eases insertion of a CE by conventional endoscopy.^3^ We present a case where an AdvanCE CE delivery device (US Endoscopy, Mentor, OH, USA) was used because the patient's small bowel could not be observed by CE alone, due to waterfall stomach.

## Case report

**FIGURE 1:**
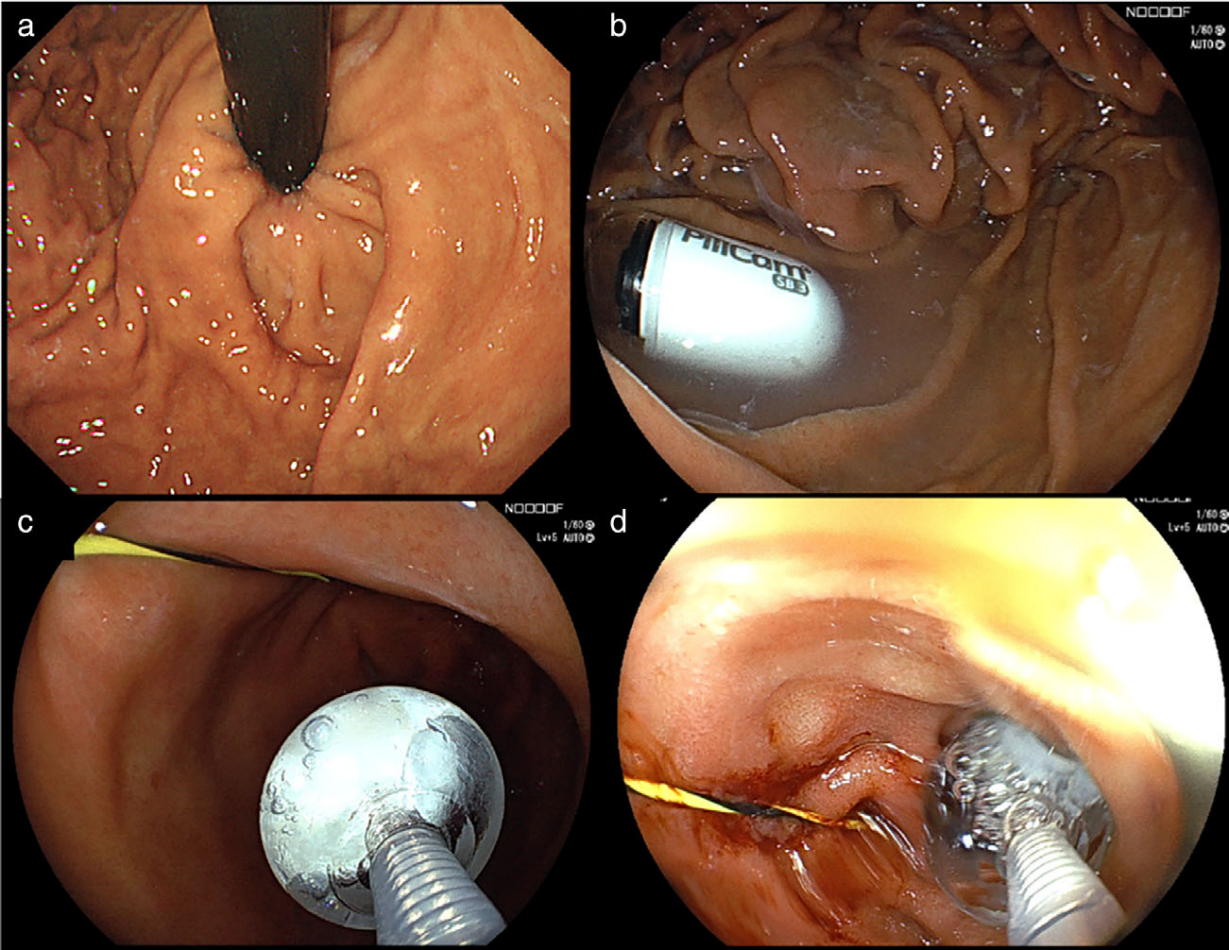
(a) Waterfall stomach. (b) Capsule endoscopy; the capsule has become stuck in the stomach. (c) Guidewire was placed in the duodenum and inserted using the AdvanCE, using it as a guide. (d) Capsule endoscope was successfully inserted into the duodenum.

The patient had iron deficiency anemia of an unknown cause and underwent upper and lower endoscopies at another hospital with no abnormal findings. CE was performed to examine the small bowel. The capsule remained stagnant in the stomach due to waterfall stomach (Fig. 1a,b). After swallowing a capsule, an endoscope was used to guide the capsule to the duodenum by grasping it with a net and a snare, but these attempts were unsuccessful due to the patient's waterfall stomach. The capsule could not be inserted into the duodenum using a net because it was not rigid enough and insufficient force was transmitted to the scope tip.

The axis of the capsule could not be aligned with the pyloric ring using a snare, and it ultimately slipped. As the direction of travel was unknown (Fig. 1c), a guidewire (GW) was placed and the capsule was grasped using AdvanCE, to guide it into the duodenum for implantation (Fig. 1d).

## Discussion

The axis of AdvanCE can be aligned with an endoscope, and its hardness allows more force to be transmitted to the tip. This rigid capsule is suitable for patients who cannot pass capsules through the pylorus. In such cases, practitioners can guide CEs into the duodenum using AdvanCE, instead of by snare/net.
